# Electromagnetic–Thermal Coupling Competition in Ag@TiO_2_ Core–Shell Nanorods Under Distance-Dependent Interaction

**DOI:** 10.3390/nano16140837

**Published:** 2026-07-08

**Authors:** Bojun Pu, Paerhatijiang Tuersun, Jingxian Wang, Guoming He, Fengyi Dou, Ye Zheng

**Affiliations:** Xinjiang Key Laboratory of Luminescence Minerals and Optical Functional Materials, School of Physics and Electronic Engineering, Xinjiang Normal University, Urumqi 830054, China; 17716902615@163.com (B.P.); 107622024210497@stu.xjnu.edu.cn (J.W.); 13769520703@163.com (G.H.); 15517338303@163.com (F.D.); zhengyejiayou@163.com (Y.Z.)

**Keywords:** core-shell nanoparticles, localized surface plasmon resonance, photothermal coupling effect, photothermal field distribution, finite element method

## Abstract

The photothermal response of plasmonic nanomaterials is strongly affected by the electromagnetic and thermal interactions between neighboring particles. In this work, the finite element method was employed to investigate the distance-dependent photothermal behavior of Ag@TiO_2_ core–shell nanorods under 808 nm laser irradiation. A single-nanorod model was first used to analyze the optical absorption and temperature distribution of an isolated nanorod, and an idealized two-nanorod model was then established to examine the coupled electromagnetic and thermal fields at different inter-particle spacings. The results show that reducing the inter-particle distance induces two competing effects: thermal-field superposition enhances local heat accumulation, whereas strong near-field plasmonic coupling at small separations modifies the electromagnetic field distribution, induces resonance shifts, and reduces the effective absorption cross-section, thereby weakening heat generation. Consequently, the temperature response exhibits a non-monotonic dependence on inter-particle distance, reflecting the competition between thermal-field overlap and plasmonic coupling. This work helps clarify the electromagnetic–thermal coupling mechanism of Ag@TiO_2_ core–shell nanorods under near-infrared irradiation and provides a theoretical reference for understanding their distance-dependent photothermal response.

## 1. Introduction

Photothermal therapy has emerged as a promising strategy for cancer treatment because of its minimal invasiveness, high efficiency, and relatively low side effects [1,2,3,4,5]. In this approach, photothermal agents absorb incident light and convert it into heat through non-radiative relaxation processes, thereby inducing localized hyperthermia for tumor ablation. Among various photothermal materials, plasmonic nanoparticles have attracted extensive attention because they can support localized surface plasmon resonance (LSPR), which enables strong light absorption at specific wavelengths and efficient photothermal conversion [6,7,8,9]. A variety of nanomaterials, including gold [10,11], silver [12,13], bismuth [14], carbon-based nanostructures [15,16,17,18], and silica-coated core–shell nanostructures [19], have been widely investigated for photothermal applications. In recent years, metal–semiconductor composite nanomaterials have become an active research topic in photothermal therapy due to their tunable optical properties, structural versatility, and potential biocompatibility [7,20,21,22].

Among these materials, Ag nanoparticles are particularly attractive because of their strong LSPR response and lower cost compared with Au-based nanostructures. However, the potential biological toxicity of Ag remains a critical concern. Ag can interact with cell membranes, leading to membrane damage, permeability changes, leakage of intracellular contents, and disruption of cellular metabolism [23]. In acidic intracellular environments such as lysosomes, Ag may also dissolve and release Ag^+^ ions, which can bind to thiol-containing biomolecules, deplete antioxidant defenses, and intensify oxidative stress and protein denaturation [24]. To reduce these risks and improve material stability, coating Ag with a TiO_2_ shell is a reasonable strategy. The TiO_2_ shell can act as a protective barrier to reduce direct exposure of the Ag core to the biological environment, while also improving the chemical stability and dispersion of the nanostructure. In addition, TiO_2_-based shells provide a representative platform for studying the electromagnetic–thermal behavior of metal–semiconductor core–shell systems.

Compared with other commonly used biocompatible oxide coatings, such as SiO_2_, Al_2_O_3_, and ZrO_2_, TiO_2_ offers several additional advantages for Ag-based photothermal nanostructures. TiO_2_ possesses good chemical stability, biocompatibility, and corrosion resistance, which can reduce the direct exposure of the Ag core to the biological environment and partially suppress Ag^+^ release [25]. Its relatively high refractive index can also modulate the local dielectric environment around the Ag core and tune the localized surface plasmon resonance [26,27]. More importantly, TiO_2_ is not merely a passive protective shell; as a semiconductor, it exhibits photocatalytic activity and potential reactive oxygen species generation capability under suitable excitation conditions, which may provide opportunities for photothermal–photocatalytic synergistic therapy. In addition, TiO_2_ surfaces contain abundant hydroxyl groups and can be readily modified with polymers, biomolecules, or targeting ligands, making them suitable for biomedical functionalization [28,29]. TiO_2_ may act not only as an optical dielectric shell but also as a thermal transport layer between the Ag core and surrounding medium. Because the thermal conductivity of TiO_2_ is lower than that of Ag, it may influence heat dissipation and local temperature gradients. TiO_2_ is also abundant, low-cost, and compatible with mature synthetic routes such as sol–gel and hydrothermal methods [30]. These features make TiO_2_ a functional shell material rather than only a passive coating.

Ag/TiO_2_-based nanostructures have been extensively studied as typical noble-metal/semiconductor hybrid systems. In these structures, Ag provides strong localized surface plasmon resonance and efficient light absorption, whereas TiO_2_ improves stability, reduces direct Ag exposure, modulates the dielectric environment, and offers additional semiconductor-related functionality. Various Ag/TiO_2_ architectures, including Ag-decorated TiO_2_ nanoparticles, Ag/TiO_2_ composites, Ag@TiO_2_ core–shell particles, and anisotropic Ag@TiO_2_ nanorods or nanospheroids, have been reported for photocatalysis, antibacterial treatment, bioimaging, and photothermal-related applications [31,32,33,34,35]. Their optical and thermal responses are strongly affected by morphology, shell thickness, dielectric environment and Ag–TiO_2_ interfacial interactions.

In our previous works [29,31], by combining the LSPR effect of the Ag core with the chemical stability and biological safety provided by the TiO_2_ shell, the optical absorption characteristics of Ag@TiO_2_ core–shell nanorods were systematically investigated, and their structural parameters for photothermal applications at 808 nm and 1064 nm were optimized. The results showed that, when the TiO_2_ shell thickness was fixed at 10 nm, Ag@TiO_2_ core–shell nanorods with a length of 62 nm and an aspect ratio of 2.8 exhibited the strongest optical absorption under 808 nm irradiation. In addition, our previous work on Ag@TiO_2_ core–shell nanostructures discussed the influence of orientation angle on plasmonic mode evolution. For nanorod systems, the plasmonic response can similarly be divided into transverse and longitudinal modes, among which the longitudinal mode generally produces stronger optical absorption and scattering. Since photothermal performance is closely related to optical absorption, selecting the strongest-absorption excitation configuration as a benchmark provides a clear and rational starting point for analyzing the effect of particle temperature-rise behavior.

For photothermal applications, Ag@TiO_2_ core–shell nanostructures are attractive because the plasmonic Ag core can efficiently convert incident light into heat, while the TiO_2_ shell can improve stability, provide a modifiable surface, and tune the local optical field. However, photothermal heating is determined not only by the intrinsic absorption of individual particles, but also by their local concentration and spatial distribution [36]. The concentration is mainly determined by the distance between the particles. At low concentrations (where the particle distance is large), the insufficient overlap of the thermal field limits the temperature increase; while at high concentrations (where the particle distance is small), the enhanced coupling of plasmons between particles may induce resonance shifts and reduce the absorption of the excitation wavelength, thereby inhibiting further heating [37,38,39]. Therefore, the photothermal response does not monotonically increase with the distance between the particles but is controlled by the competition between thermal superposition and plasmon coupling. Although previous studies have mainly addressed the synthesis, optical optimization, photocatalytic performance, antibacterial activity, and overall photothermal conversion of Ag/TiO_2_ nanostructures [29,30,31,40], this concentration-dependent behavior remains insufficiently understood. Therefore, Ag@TiO_2_ core–shell nanorods are used here as a representative plasmonic metal/semiconductor model system, rather than a universally optimal platform, to investigate the competitive relationship of photothermal coupling under ideal electromagnetic–thermal simulation conditions.

In this work, Ag@TiO_2_ core–shell nanorods were selected as a representative model system, and finite element simulations were employed to investigate their distance-dependent photothermal behavior under 808 nm laser irradiation. A single-nanorod model was first established to analyze the optical absorption and temperature distribution of an isolated nanorod, followed by an idealized two-nanorod model to examine the coupled electromagnetic and thermal fields at different inter-particle spacings. In photothermal therapy, nanoparticle concentration is closely related to the average inter-particle distance, which can simultaneously affect thermal-field overlap and plasmonic coupling. A smaller inter-particle distance may promote local heat accumulation through thermal-field superposition, but excessive proximity can also alter the electromagnetic field distribution, induce resonance shifts, and reduce effective optical absorption. Thus, the concentration-dependent photothermal response is expected to be constrained by the competition between these two mechanisms. The purpose of this study is to clarify this competing electromagnetic–thermal coupling mechanism in Ag@TiO_2_ core–shell nanorods and to provide a theoretical reference for understanding the role of particle concentration in photothermal therapy-related studies.

## 2. Model and Method

### 2.1. Simulation Model

**Figure 1:**
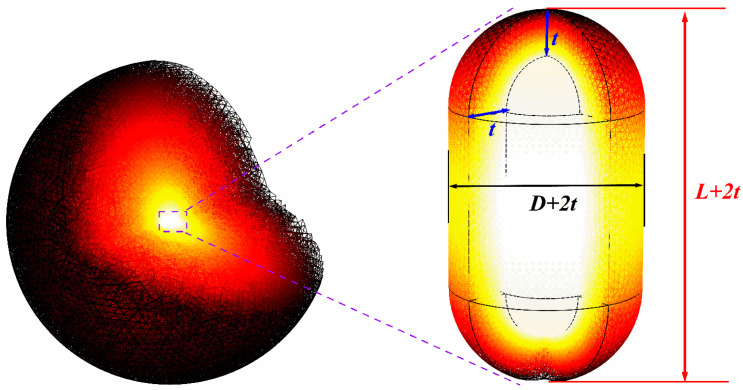
Schematic diagram of the simulation area and the model of Ag@TiO_2_. *L* represents the length of the core Ag, *t* represents the thickness of the shell TiO_2_, and *D* represents the diameter of the core. The incident light wavelength is 808 nm, and the refractive index of the SM is 1.44.

A three-dimensional finite element method [41,42,43] was adopted to simulate the photothermal response of single and double Ag@TiO_2_ core–shell nanorods under 808 nm near-infrared laser irradiation. An electromagnetic field–heat conduction unidirectional coupling model was constructed to accurately calculate the local temperature distribution of the nanorods at different spacings. The laser power density was set at 2.5 × 10^4^ W/cm^2^. The optical parameters of the core material Ag were input based on the measured values from Johnson and Christy [44], and the optical parameters of the shell TiO_2_ were input based on the measured values from Sarkar et al. [45]. The geometric dimensions are shown in Figure 1. The core length *L* was set to 62 nm, the diameter *D* was *L*/*AR*, the aspect ratio *AR* was 2.8, and the shell thickness *t* was 10 nm. The model domain was set as the surrounding tissue, with a surrounding medium (SM) refractive index of 1.44 and a radius of *λ*/2 + (*L* + *D* + 2*t*)/2. A perfect matching layer boundary condition with a thickness of *λ*/4 was set outside the surrounding tissue to ensure that the electromagnetic waves were completely absorbed, thus equivalent to an infinitely large region.

During the solution process of the electromagnetic field, the finite element method is used to solve the Helmholtz equation to calculate the absorption cross-section of the nanorod under the irradiation of 808 nm laser. Subsequently, the Joule heat is taken as the heat source term and substituted into the heat conduction equation. Combined with the finite element method, the heat conduction equation is solved, and finally, the heat field distribution is obtained. The model boundary is set as a constant temperature boundary condition, and the initial environmental temperature is set at 310 K to simulate the process of heat transfer to an infinitely large environment. The heat transfer parameters used in this paper are from [46] as shown in Table 1.

### 2.2. Analytical Method for Nanosphere Heating Calculation

During the interaction between light and metal nanoparticles, due to the existence of the imaginary part of the dielectric function of the metal nanoparticles, the metal nanoparticles exhibit an absorption effect on light. To quantify the absorption capacity of the metal nanoparticles on light, the absorption cross-section (*C*_abs_) can be used for description. The absorption cross-section is defined as the ratio of the absorbed light energy (*W*_a_) of the metal nanoparticles to the incident irradiance (*I*_i_):

(1) $$C_{\mathrm{abs}}=\frac{W_{a}}{I_{i}},$$

where the incident irradiance *I*_i_ = (*n*_m_ *E*^2^)/(2*Z*_0_), *n*_m_ is the refractive index of the SM, *E* is the amplitude of the incident electric field, and *Z*_0_ is the wave impedance in a vacuum. The electrons inside the metal sphere absorb the energy of the photons and undergo intense oscillations within. During the electron oscillation process, due to the existence of resistance, electrons collide with ionized matter, resulting in energy dissipation in the form of Joule heat, thereby causing a local temperature increase in the nanosphere. The process of converting the kinetic energy of electrons into heat energy is regarded as a steady-state heat source, and its heat power density [47] is expressed as *P*_abs_ = *I*_i_·*C*_abs_. Based on the simultaneous solution of the Poisson equation and Fourier’s law of heat conduction, the temperature variation at a position with a distance of *r* from the center of the sphere in the external environment of a nanosphere with a radius of *a* over time can be obtained [48].

(2) $$T(r,t)=T_{0}+\frac{P_{\mathrm{abs}}}{4k\pi ar}erfc(\frac{r-a}{2\sqrt{\alpha t}})\;(r>a),$$

where *k* represents the thermal conductivity of the medium, *α* = *k*/*ρc*_p_ represents the thermal diffusivity, *ρ* is the density of the nanospheres, *c*_p_ is the constant-pressure heat capacity, and *erfc* is the complementary error function. This analytical model assumes that the energy is uniformly distributed on the surface of the sphere and ignores convective heat dissipation. It is applicable for near-field thermal analysis of small-sized nanoparticles in a low Reynolds number environment.

## 3. Results and Discussion

### 3.1. Method Validation

**Figure 2:**
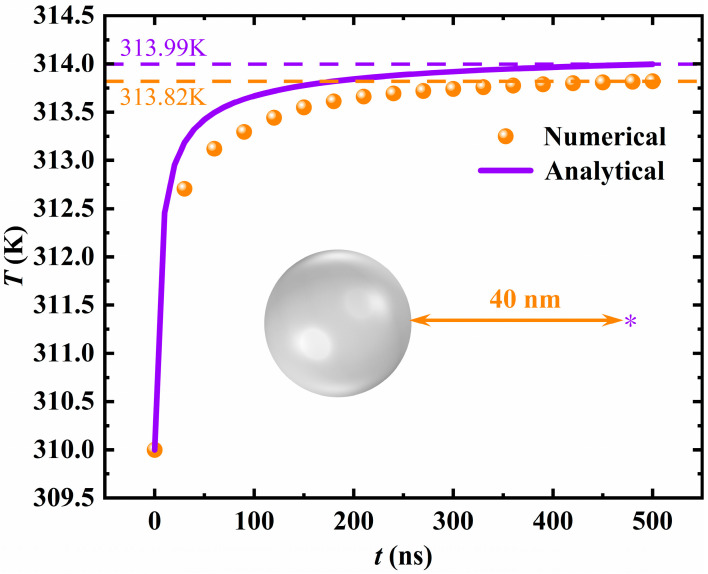
The variation in temperature at a point 40 nm (the position marked by “*” in the figure) away from the center of an Ag nanosphere with a radius of 10 nm along the outer surface under the two calculation methods. The incident light wavelength is 808 nm, the refractive index of the SM is 1.44, the incident light intensity is 2.5 × 10^4^ W/cm^2^, and the initial temperature is 310 K.

Figure 2 shows the variation in temperature at a point 40 nm away from the center of an Ag nanosphere with a radius of 10 nm over time, simulated using both the finite element method and the analytical method. The incident light wavelength is 808 nm, the SM refractive index is 1.44, the incident light intensity is 2.5 × 10^4^ W/cm^2^, and the initial temperature is 310 K. During the finite element method calculation, independent tests were conducted on the mesh division density and time step to ensure numerical convergence. By comparing the temperature responses under different boundary conditions, it was confirmed that the constant temperature boundary condition is equivalent to the simulation of an infinite area for the local thermal field. From the figure, it can be seen that the temperature rapidly rises within a short period of time and reaches a stable state within approximately 500 ns. The calculation results of the two methods are highly consistent, verifying the reliability of the model. The error between the finite element method and the analytical solution is less than 0.05%, indicating that the established finite element model can accurately simulate the transient temperature evolution behavior around the nanoparticles during the photothermal conversion process. It can accurately predict the thermal field distribution around the nanoparticles, laying a foundation for subsequent simulation results.

### 3.2. Heating Range of Ag@TiO_2_ Nanorods

**Figure 3:**
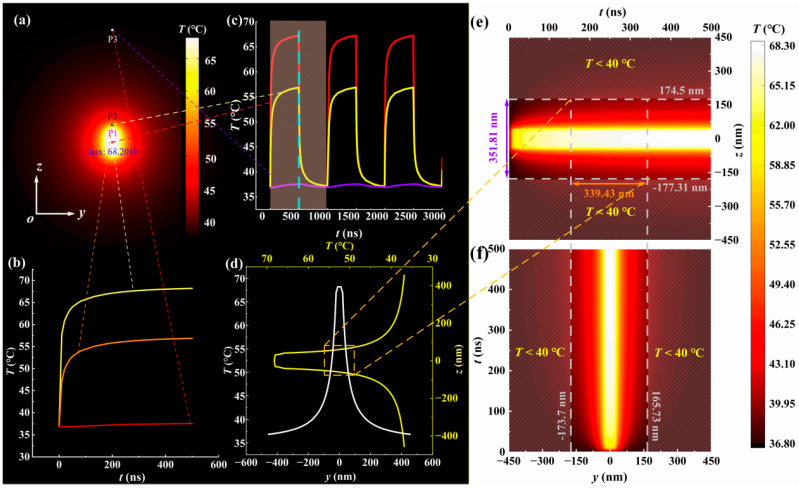
(**a**) Temperature distribution around Ag@TiO_2_ core–shell nanorods at 500 ns; (**b**) At the center point P_1_ (0, 0, 0) of the nanorods, point P_2_ (0, 0, *L*/2 + 2*d*) near the outer long axis direction of the nanorods away from the rod, and point P_3_ (0, 0, *L*/2 + 30*d*) at the outer long axis direction of the nanorods closer to the rod within 500 ns, the temperature changes with time when the incident light wavelength is 808 nm, the SM refractive index is 1.44, the incident light intensity is 2.5 × 10^4^ W/cm^2^, and the initial temperature is 310 K; (**c**) The temperature changes with time at points P_1_, P_2_, and P_3_ under pulsed illumination with a period of 500 ns; (**d**) Temperature distribution of the nanoparticles along the *y*-axis (white) and the *z*-axis (yellow) passing through point P_1_ at 500 ns; (**e**,**f**) Show the temperature rise of the two lines in (**d**) with time changes. In all simulations, the incident optical vector oscillation direction is along the *z*-axis (particle long axis).

The advantage of the finite element method lies in its ability to calculate nanoparticles of any shape. Thus, Ag nanospheres can be freely transformed into Ag@TiO_2_ core–shell nanorods, allowing for further exploration of the threshold concentration required for nanorods to participate in photothermal therapy. Figure 3 shows the range that a single Ag@TiO_2_ nanorod can affect when the temperature reaches a steady state. Figure 3b first selects three representative points: the center point P_1_ (0, 0, 0) of the rod, a point P_2_ (0, 0, *L*/2 + 2*d*) close to the rod along the rod’s length axis in the external environment, and a point P_3_ (0, 0, *L*/2 + 30*d*) far from the rod along the rod’s length axis in the external environment. By refining the temperature increment step, the time for the temperature to reach a steady state is finally determined to be around 500 ns. Then, the incident light is pulsed modulated at a 500 ns period to prevent the accumulation of heat from continuous illumination from damaging the surrounding normal tissues. Under pulsed illumination, the temperature change shows a periodic rise and fall, demonstrating good thermal response controllability. To obtain the range of the environment affected by the heat radiated by a single nanorod, we discussed the temperature along the straight lines passing through point P_1_ along the *y*-axis and *z*-axis, as shown in Figure 3d. From the figure, it can be seen that the temperature near the rod is the highest, and the heat energy is mainly concentrated in the near-field region of the nanorod. The temperature gradually decays exponentially from the center of the rod to the outside. As the distance increases, the temperature gradient gradually flattens, indicating that the thermal diffusion range is limited, and the effective action area is concentrated within a few hundred nanometers around the nanorod. Figure 3e,f further show that the temperature rise process along the *y*-axis and *z*-axis gradually expands over time. The initial expansion range of the temperature increase is rapid, and then gradually converges to the steady-state temperature range at 500 ns, reflecting the balance of heat conduction accumulation and dissipation in the medium. We selected the range where the temperature exceeds 40 °C as the effective photothermal action area (the normal temperature range should be 50–60 °C, considering the thermal coupling between particles, here we first select a roughly defined range). The maximum thermal diffusion distance along the *z*-axis is approximately 351.81 nm, and along the *y*-axis it is 339.43 nm. The thermal diffusion along the long axis is slightly greater than that along the short axis, indicating that this range is related to the geometric anisotropy of the nanorod.

**Figure 4:**
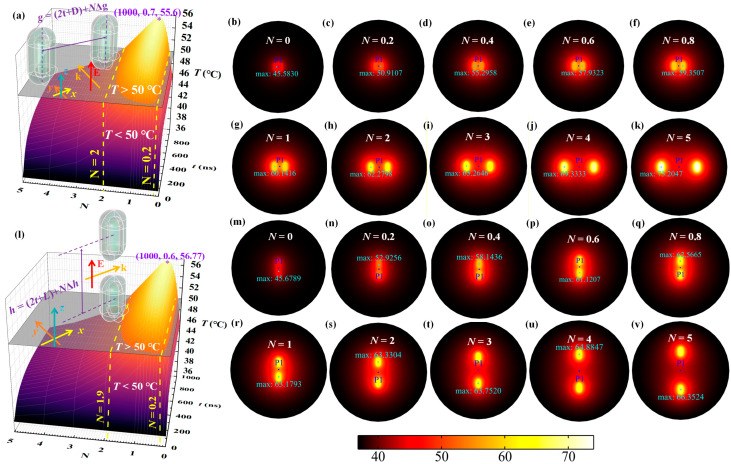
When two rods are arranged side by side (**a**) and when they are arranged end to end (**l**), a point P_1_ (0, 0, 0) is taken in the middle between the two rods. The temperature distribution of P_1_ as a function of rod spacing and time (the “*” in the figure indicates the position of the maximum value.); (**b**–**k**) The temperature distribution on the *yoz* plane of the side-by-side arranged nanorods during the steady state of 1000 ns. (**m**–**v**) The temperature distribution on the *yoz* plane of the end-to-end arranged nanorods during the steady state of 1000 ns, as a function of rod spacing. In the above simulations, the incident light wavelength is 808 nm, the refractive index of the SM is 1.44, the incident light intensity is 2.5 × 10^4^ W/cm^2^, and the initial temperature is 310 K; the incident optical vector oscillation direction is all along the *z*-axis (the long axis of the particle).

Taking into account the thermal coupling effect between particles in practical applications, that is, the superposition of thermal fields under dense distribution conditions, in order to further determine the critical concentration of a single nanorod to achieve overall efficient thermal ablation in the tumor area, we selected two nanorods to study their photothermal coupling effect when they are arranged side by side [see Figure 4a]. The distance between them is *g* = (2*t* + *D*) + *N*Δ*g*, where Δ*g* = [339.43 nm − (2*t* + *D*)]/5, and *N* ranges from 0 to 5 (with a step size of 0.2 from 0 to 1 and 1 to 5). When they are arranged end-to-end [see Figure 4l], the distance between them is *h* = (2*t* + *L*) + *N*Δ*h*, where Δ*h* = [351.81 nm − (2*t* + *L*)]/5, and *N* ranges from 0 to 5 (with a step size of 0.2 from 0 to 1 and 1 to 5). *t* is the thickness of the TiO_2_ shell layer, *D* is the diameter of the Ag rod, the electric field oscillation direction is along the *z*-axis (long axis), the incident light wavelength is 808 nm, the SM refractive index is 1.44, and the incident light intensity is 2.5 × 10^4^ W/cm^2^. The calculation results show that when *N* ≤ 0.7, the temperature gradient is large and the temperature drop is significant; when *N* > 0.7, the heating gradient gradually decreases and the temperature drop trend becomes more gentle. At *N* = 0.7, the temperature reaches its maximum value of 55.6 °C; while the end-to-end arrangement also exhibits a similar pattern, at *N* = 0.6, the temperature reaches its maximum value of 56.77 °C. In the side-by-side arrangement mode, when 0.2 ≤ *g* ≤ 2, the steady-state temperature at point P_1_ exceeds 50 °C, while in the end-to-end arrangement mode, when 0.2 ≤ *h* ≤ 1.9, the same phenomenon is observed.

**Figure 5:**
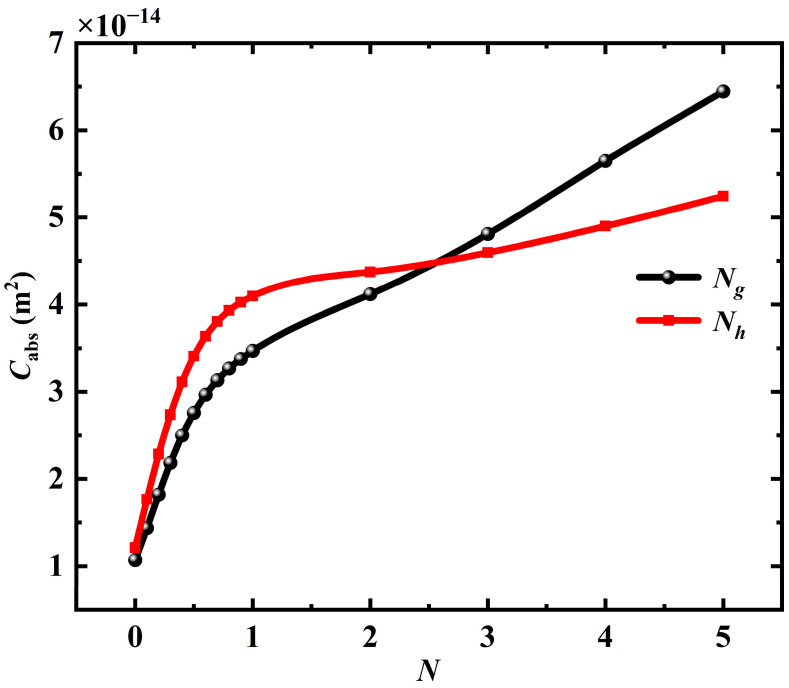
The variation in the absorption cross-section of the overall two Ag@TiO_2_ core–shell nanorods with the particle spacing. The black line represents the spacing change when arranged side by side, and the red line represents the spacing change when arranged end to end.

Figure 4b–k,m–v show the distribution of the temperature field of the nanorods under different arrangement methods. It is not difficult to observe from the figures that it is not the case that the smaller the distance between the two rods, the higher the temperature. Instead, there exists an optimal coupling distance. When the distance between the side-by-side nanorods is too close, the superposition effect of the local electromagnetic field is enhanced, and the local electric field will cause its resonance peak to shift towards the blue. The reason is that for the nanorods arranged side by side, when a beam of light shines on two rods simultaneously, the same electric field causes the internal electrons of both rods to vibrate synchronously. When the distance between the two rods is close, they are both in the local electric field outside each other. The closer the rods are, the denser the electric field lines are and the stronger the local electric field is. The external local electric field generated by one rod will promote the oscillation of the free electrons inside the other rod, thereby accelerating its oscillation frequency, resulting in an increase in the matching incident light frequency, and thus causing the resonance peak to shift towards the blue direction. On the contrary, when two rods are arranged head to head, the situation is the opposite. When the distance between the two rods is too close, the local electric fields generated by the two rods have an inhibitory effect on the oscillation of the internal electrons of each other, causing a decrease in frequency and moving the resonance peak towards the red direction. Due to the movement of the resonance peak, even without changing other conditions, the difference in the maximum temperature rise can be very large. As shown in Figure 5, this is when irradiated by an 808nm laser, the absorption cross-section of the two rods changes with the distance between the nanorods. From the figure, it can be seen that whether it is the side-by-side spacing or the head-to-head spacing, during the process of its reduction, the absorption cross-section gradually decreases, and when *N_g_* and *N_h_* are less than 1, the absorption cross-section decreases even faster. In the process of reducing *N_g_* and *N_h_* from 5 to 0 in this paper, the absorption cross-section decreased by about 5–6 times. The shift of the resonance peak of the nanorods directly affects the absorption efficiency of the nanorods for light energy, thereby determining the thermal conversion efficiency. When the distance between the nanorods is too far, the absorption cross-section increases gradually and approaches twice the absorption cross-section of a single particle, at which point the absorption cross-section remains unchanged but the particle spacing is relatively large, resulting in a decrease in the heat coupling ability and a limited distance for heat propagation, and the surrounding large area does not reach the requirements of the treatment temperature. Therefore, in order to achieve photothermal therapy at the desired temperature, the spacing between the nanorods needs to be precisely controlled within the range of 0.2 ≤ *N_g_* ≤ 2 and 0.2 ≤ *N_h_* ≤ 1.9, that is, the spacing between the rods should be controlled within 54.03 nm ≤ *g* ≤ 161.06 nm and 93.89 nm ≤ *h* ≤ 194.97 nm to achieve efficient thermal ablation in the lesion area.

## 4. Conclusions

In conclusion, the photothermal behavior of Ag@TiO_2_ core–shell nanorods was investigated using finite element simulations. A single-nanorod model was first used to analyze the optical absorption and temperature distribution of an isolated nanorod, and an idealized two-nanorod model was then established to examine the coupled electromagnetic and thermal fields at different inter-particle spacings. The results show that the photothermal response of Ag@TiO_2_ core–shell nanorods is strongly dependent on inter-particle distance. As the distance between neighboring nanorods decreases, thermal-field superposition enhances local heat accumulation and promotes temperature rise. However, excessive spatial proximity strengthens near-field plasmonic coupling, which can modify the electromagnetic field distribution, induce resonance shifts, reduce the effective absorption cross-section, and consequently weaken heat generation. Therefore, the temperature response exhibits a non-monotonic dependence on inter-particle spacing, reflecting the competition between thermal-field overlap and plasmonic-coupling-induced absorption modulation. From the perspective of photothermal therapy-related studies, the inter-particle distance between neighboring nanorods plays a key role in regulating both thermal-field overlap and optical absorption. Within the present model framework, under 808 nm laser irradiation, a surrounding medium refractive index of 1.44, and the strongest absorption configuration in which the incident polarization is parallel to the nanorod long axis, the favorable coupling distance was determined to be 54.03–161.06 nm for side-by-side nanorods and 93.89–194.97 nm for head-to-head nanorods. These ranges arise from the balance between thermal-field superposition and plasmonic-coupling-induced absorption reduction. Excessively small spacing leads to strong near-field coupling, resonance shifts, and a reduced absorption cross-section, whereas excessively large spacing weakens thermal coupling between nanorods. Therefore, the obtained results should be regarded as favorable inter-particle spacing ranges under idealized simulation conditions, rather than as concentration windows or therapeutic dosage criteria. It should be noted that the present simulations were performed under simplified assumptions, including ideal dispersion, homogeneous surrounding conditions, and fixed particle orientation corresponding to the strongest absorption configuration. In realistic biological environments, factors such as random particle orientation, aggregation, and tissue heterogeneity may affect the electromagnetic and thermal interactions between particles and alter the actual photothermal response. In addition, the crystalline phase of TiO_2_ may influence its optical properties and the plasmonic response of Ag@TiO_2_ nanorods, which was not explicitly considered in the present model. Future work will further incorporate these factors to improve the applicability of the model under more realistic conditions.

## Figures and Tables

**Table 1:** Heat transfer parameters of Ag, TiO_2_, and Cancer Tissue [46].

Material	Density (*ρ* [kg/m^3^])	Specific Heat Capacity (*C*_p_ [J/kg/K])	Thermal Conductivity (*k* [W/(m·K)])
Ag	10,500	235	429
TiO_2_	3800	686	6.7
Cancer Tissue	997	4182	0.6

## Data Availability

The original contributions presented in this study are included in the article; further inquiries can be directed to the corresponding author.

## Abbreviations

The following abbreviations are used in this manuscript:

LSPR	localized surface plasmon resonance
FEM	finite element method
SM	surrounding medium
